# The Role of Altered BDNF/TrkB Signaling in Amyotrophic Lateral Sclerosis

**DOI:** 10.3389/fncel.2019.00368

**Published:** 2019-08-13

**Authors:** Jonu Pradhan, Peter G. Noakes, Mark C. Bellingham

**Affiliations:** ^1^Faculty of Medicine, School of Biomedical Sciences, The University of Queensland, Brisbane, QLD, Australia; ^2^Queensland Brain Institute, The University of Queensland, Brisbane, QLD, Australia

**Keywords:** BDNF, TrkB receptors, A_2__*a*_R, motor neurons, ALS, MND

## Abstract

Brain derived neurotrophic factor (BDNF) is well recognized for its neuroprotective functions, via activation of its high affinity receptor, tropomysin related kinase B (TrkB). In addition, BDNF/TrkB neuroprotective functions can also be elicited indirectly via activation of adenosine 2A receptors (A_2__*a*_Rs), which in turn transactivates TrkB. Evidence suggests that alterations in BDNF/TrkB, including TrkB transactivation by A_2__*a*_Rs, can occur in several neurodegenerative diseases, including amyotrophic lateral sclerosis (ALS). Although enhancing BDNF has been a major goal for protection of dying motor neurons (MNs), this has not been successful. Indeed, there is emerging *in vitro* and *in vivo* evidence suggesting that an upregulation of BDNF/TrkB can cause detrimental effects on MNs, making them more vulnerable to pathophysiological insults. For example, in ALS, early synaptic hyper-excitability of MNs is thought to enhance BDNF-mediated signaling, thereby causing glutamate excitotoxicity, and ultimately MN death. Moreover, direct inhibition of TrkB and A_2__*a*_Rs has been shown to protect MNs from these pathophysiological insults, suggesting that modulation of BDNF/TrkB and/or A_2__*a*_Rs receptors may be important in early disease pathogenesis in ALS. This review highlights the relevance of pathophysiological actions of BDNF/TrkB under certain circumstances, so that manipulation of BDNF/TrkB and A_2__*a*_Rs may give rise to alternate neuroprotective therapeutic strategies in the treatment of neural diseases such as ALS.

## Introduction

Amyotrophic lateral sclerosis (ALS), the most common form of motor neuron disease (MND), is a fatal adult onset neurodegenerative disease resulting in progressive and preferential degeneration and death of upper motor neurons (UMNs, corticospinal neurons) of the motor cortex, and alpha lower motor neurons (LMNs) of the brain stem and the spinal cord (Cleveland and Rothstein, 2001; Turner et al., 2013). The incidence of ALS is 1.7 per 100,000 people each year (Pasinelli and Brown, 2006; Marin et al., 2017; Sandstedt et al., 2018). Only 10% of all ALS cases exhibit familial inheritance (fALS) (Turner et al., 2013) while the remaining 90% are sporadic (sALS). Mutations in the gene encoding Cu/Zn superoxide dismutase 1 (SOD1) were the first to be identified as a primary ALS mutation (Rosen et al., 1993) and have been also the most characterized, with several widely used mouse models of SOD1 mutations (Gurney et al., 1994). Overall, SOD1 mutations account for 20% of fALS and 1–2% of sALS, with more than 180 mutations identified within the SOD1 gene (Hayashi et al., 2016).

**FIGURE 1:**
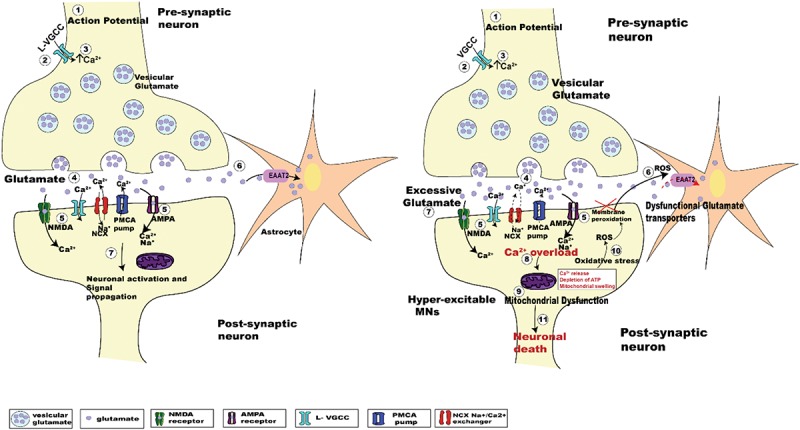
Schematic diagrams showing the regulation of glutamatergic synaptic transmission at upper and lower MN synapses in normal and SOD1^*G*93*A*^ mice. The arrival of the action potential **(step 1)**, triggers the influx of Ca^2+^ ions into pre-synaptic terminal **(step 2)**, the rise in intracellular calcium **(step 3)** in turn triggers the fusion of synaptic vesicles with the pre-synaptic membrane to release glutamate (light purple dots) from the terminal **(step 4)**. Binding of glutamate to its postsynaptic receptors (NMDA, green; and AMPA, magenta; **step 5**), leads to the influx of Ca^2+^ ions. During this process, concentration of glutamate within the synaptic cleft is reduced by the uptake of glutamate into Astrocytes (orange star shaped cells) via EAAT2 glutamate transporters (pink; **step 6**). Within the post-synaptic neuron the influx of Ca^2+^ via glutamatergic receptors plus voltage gated Ca^2+^ channels, along with an influx of sodium (Na^+^) ions lead to the activation of the postsynaptic neuron **(step 7)**. The subsequent lowering of Ca^2+^ post-synaptic transmission is managed by extrusion of calcium via ATP pumps (PMCA pumps) and Na^+^/Ca^2+^ exchanger (NCX), plus calcium uptake into intracellular stores (ER and mitochondria) (Chio et al., 2012). The right panel shows the pathogenesis of glutamate induced excitotoxicity in ALS. Excessive glutamate released in the synaptic cleft triggers increased activation of the post-synaptic glutamate receptors (NMDA and AMPA receptors). This effect is enhanced due to dysfunctional glutamate transporters (EAAT2; **step 6**, *right panel*, red dashed arrow), which lengthen the persistence of glutamate within the synaptic cleft (**step 7**, *right panel*), which further activates glutamatergic receptors. Impaired Na^+^/Ca^2+^ exchanger and ATP pumps in SOD1^*G*93*A*^ mice results in enhanced Ca^2+^ intracellularly (DeJesus-Hernandez et al., 2011; Sirabella et al., 2018). Generation of reactive oxygen species (ROS) causes neuronal membrane peri-oxidation impairing the glutamate transporters (EAAT2). The enhanced activation of these receptors leads to increased Ca^2+^ overload (**step 8**, *right panel*) in the post-synaptic neuron, which in turn leads to mitochondrial dysfunction (**step 9**, *right panel*), oxidative stress (**step 10**, *right panel*), and generation of reactive oxygen species (ROS) ultimately leading to motor neuron death (**step 11**, *right panel*).

Despite decades of research, the pathogenic mechanism underlying death of UMNs and LMNs is still unclear. Numerous etiologies have been proposed, including oxidative stress, mitochondrial dysfunction, protein aggregation, RNA processing, autophagy, and glutamate excitotoxicity (Chico et al., 2017). Glutamate excitotoxicity, the focus of this review, results from a disruption of the finely tuned cellular response to input stimuli, resulting in excessive glutamate release from the pre-synaptic neuron, delayed clearance from the synaptic cleft or increased responsiveness by glutamate receptors post-synaptically (Rothstein et al., 1992; Hayashi et al., 2016). Excessive release of glutamate induced by Ca^2+^ dysregulation within the pre-synaptic compartment (Van Den Bosch et al., 2006; King et al., 2016), causes a prolonged state of activation of postsynaptic glutamate N-methyl-D-aspartate (NMDA) and alpha-amino-3-hydroxy-5-methyl-4-isoxazole propionate (AMPA) receptors. In addition to this, the Ca^2+^ buffering capacity of MNs in ALS is weakened at an early age, with impairment of the Ca^2+^ ATPase and Na^+^/Ca^2+^ exchanger adding to the cytoplasmic Ca^2+^ load (DeJesus-Hernandez et al., 2011; Sirabella et al., 2018). Enhanced post-synaptic glutamate receptor activation is physiologically observed as synaptic hyper-activity of upper and lower MNs (van Zundert et al., 2008; Fogarty et al., 2015). Hyper-activity also raises the level of intracellular Ca^2+^ within the post-synaptic MN, potentially creating a toxic intracellular environment that can cause cell death (Le Masson et al., 2014; Figure 1).

Amyotrophic lateral sclerosis progresses relentlessly and, without effective intervention, 50% of the patients die within 3 to 5 years post-diagnosis, due to loss of their respiratory MNs (i.e., respiratory failure) (Brown and Al-Chalabi, 2017). The only FDA approved treatments so far are riluzole, which acts to reduce the release of glutamate and hence lower neuronal excitotoxicity (Bellingham, 2013b), and edaravone, an anti-oxidant compound. Unfortunately, riluzole only marginally enhances survival by a few months (Bensimon et al., 1994; Fang et al., 2018). In 2017 after more than 20 years, a second drug Radicava (edaravone) has been FDA approved to treat ALS; thus far, edaravone has also only been shown to slow the rate of clinical progression in ALS (Abe et al., 2014). This slow development of new treatments highlights the need to better understand the cellular and molecular mechanisms of ALS, so as to develop effective combination therapies to ameliorate this multi-factorial disease.

In addition to neuronal hyper-excitability in neuromotor circuits in ALS, the level of neuronal activity strongly influences the modification of neuronal circuits in the developing CNS, by stabilizing and strengthening coincident inputs and refining/removing weaker inputs (Goodman and Shatz, 1993; Stevens et al., 2007; Kutsarova et al., 2016). This developmental plasticity initially depends on the release of neurotransmitters from the pre-synaptic neuron (Andreae and Burrone, 2018), and thus factors that increase pre-synaptic activity will also increase synaptic plasticity. In ALS, upper and lower MNs in animal models of ALS have been shown to exhibit synaptic hyper-activity (van Zundert et al., 2008; Fogarty et al., 2015). In the case of lower MNs, hyperactivity of upper MNs could in turn result in enhanced glutamate release from their nerve terminal boutons at their synapses with lower MNs (Figure 1). Excessive release of glutamate from these pre-synaptic inputs onto lower MNs could lead to their death by excitotoxicity (King et al., 2016). Similar mechanisms may also operate for excitable synaptic connections made onto upper and lower MNs from other pre-motor excitatory inputs (van Zundert et al., 2008). To complicate matters, changes in neuronal circuit activity outside of what is considered a “normal physiological range” (i.e., “hyper-excitability;” Bae et al., 2013), can induce compensatory effects termed “synaptic homeostasis” (Turrigiano, 2012). For example, in SOD1^*G*93*A*^ ALS model mice, while upper MNs have been shown to be hyper-active prior to their death, these neurons display reductions in dendritic length and spine density, suggesting a homeostatic response to heightened pre-synaptic activity (Fogarty et al., 2015; Saba et al., 2016). Alternatively, these morphological reductions may simply reflect the stressed state of the neurons as it progresses to death (Fogarty et al., 2016). Together these observations suggest that abnormal neuronal activity and death of upper and lower MNs in ALS are directly linked.

What might be the mechanism(s) that links abnormal neuronal activity to neuronal death? One proposed mechanism is the activity-dependent synthesis and release of neurotrophins (McAllister et al., 1996; Du et al., 2003; Cunha et al., 2010). Neurotrophins are secreted proteins and potent regulators of neuronal development, survival, neurogenesis and synaptic plasticity (Huang and Reichardt, 2001). They have long been targeted as prospective therapeutic agents for the treatment of neurodegenerative disorders, including ALS. The neurotrophin family constitutes nerve growth factor (NGF), brain derived neurotrophic factor (BDNF), neurotrophin-3 (NT-3), and neurotrophin-4/5 (NT-4/5). Amongst these, BDNF is abundantly expressed in the developing and adult nervous system (Murer et al., 2001) and has been extensively studied for its roles in neuronal survival (e.g., MNs) (Ringholz et al., 2005; Pansarasa et al., 2018), along with its ability to increase the release of glutamate at glutamatergic synapses (Rao and Finkbeiner, 2007; Mattson, 2008). Given these proposed roles for BDNF, namely its neurotrophic and possible neurotoxic roles, it becomes apparent that regulation of BDNF could open up new therapeutic strategies in the treatment of neurodegenerative disorders. This review focuses on the biology of BDNF and its proposed neurotrophic and neurotoxic roles in the pathogenesis and treatment of ALS.

## Biology of BDNF: From Synthesis to Secretion

Brain derived neurotrophic factor is a member of the family of growth factors and was initially purified from pig brain (Barde et al., 1982). The expression of BDNF in human, rat and mouse is encoded by a single BDNF gene, whose transcription is regulated by several promoters (Sasi et al., 2017). The human BDNF gene consists of eleven 5′ untranslated (UTR) exons, compared to 9 exons found in rodents (rats and mice), and only one 3′ coding exon. These exons initiate transcription at the ATG start codon by alternate splicing to produce 17 BDNF mRNA transcripts and 9 BDNF 5′ promoters (Aid et al., 2007; Pruunsild et al., 2007). The transcription of BDNF is neuronal activity-dependent and regulated by membrane depolarization. An increase in intracellular calcium (Ca^2+^) concentration via activation of NMDA glutamate receptors or L-type voltage gated calcium channels (L-VGCC) following a depolarizing stimulus initiates transcription of the BDNF gene, predominantly at exon IV (Tao et al., 1998; Zheng et al., 2011). The promoter of BDNF exon IV contains Ca^2+^ response elements (CaRE) – CaRE1 and CaRE3, which regulate transcription (Tao et al., 1998; Hong et al., 2008; Zheng et al., 2011). Cyclic AMP responsive element binding protein (CREB), a transcription factor, binds to these CaREs, which are phosphorylated by calcium/calmodulin (CaM)-dependent protein kinases, cAMP-dependent protein kinases and MAPK, activating the promoter and resulting in Ca^2+^ dependent transcription of BDNF mRNA at exon IV (Zheng et al., 2011).

Alternate splicing terminates transcription at two alternate polyadenylation points which shift the translation sites, giving rise to two distinct BDNF mRNA populations into specific neuronal compartments, allowing spatial and temporal translocation (Pruunsild et al., 2007; Notaras and van den Buuse, 2018). The short UTR BDNF transcripts are localized in the soma and maintain basal activity-dependent BDNF production. The long UTR BDNF transcript is targeted to the dendrites and displays robust translation on neuronal activation (An et al., 2008; Lau et al., 2010). BDNF localization is mostly somatodendritic (59%) within dense core vesicles (Tongiorgi, 2008; Dieni et al., 2012) with only 29% targeted to the dendrites (Adachi et al., 2005). The specific compartmental translation of BDNF mRNA at long or short 3′ UTR is also aided by binding to numerous microRNAs such as miR-30, resulting in degradation of BDNF transcripts (Bartel, 2004; Mellios et al., 2008) and negative regulation of BDNF synaptogenesis (Shi, 2015).

**FIGURE 2:**
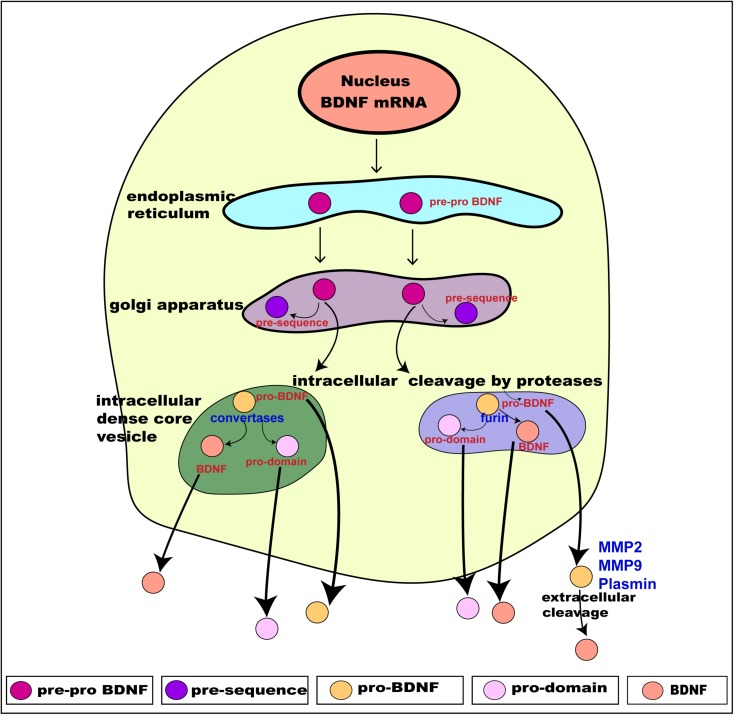
Schematic presentation of BDNF synthesis from translation, intracellular processing through to its secretion. BDNF is synthesized from the BDNF gene in a multi-step process. Intracellularly, the pre-pro BDNF is produced in the endoplasmic reticulum which is then translocated toward the Golgi apparatus, where the pre-sequence is cleaved off to form pro- BDNF. The pro-BDNF is further processed, via the Golgi apparatus, into the *trans*-Golgi network (TGN) where the pro domain is cleaved off by proteases to form mature BDNF (BDNF). The pro-BDNF is proteolytically cleaved by furin or convertase and is intracellularly secreted as BDNF. Both pro-BDNF and BDNF are preferentially grouped and packaged into secretory dense core-vesicles and secreted extracellularly via exocytosis. The extracellularly secreted pro-BDNF is then processed and catalyzed by proteases such as plasmin and metalloproteinases (MMP2 and MMP9) to form BDNF. As a result, three functionally active isoforms, namely pro-domain, pro-BDNF and BDNF are secreted extracellularly. Adapted from Kowianski et al. (2018).

Translation of these distinct alternate BDNF mRNA transcripts gives rise to the precursor pre-pro BDNF in the endoplasmic reticulum (Foltran and Diaz, 2016; Kowianski et al., 2018), consisting of a signal peptide after the initiation codon and N-glycosylation site on the pro region. It is then translocated to the Golgi apparatus, where the signal peptide pre-sequence is cleaved off to form pro-BDNF (30 kDa) (Lessmann et al., 2003). The pro-BDNF is then further processed either intracellularly or extracellularly, via the Golgi apparatus, into the *trans*-Golgi network (TGN) where the pro domain is proteolytically cleaved off to form pro-domain and mature BDNF and is secreted into the extracellular space (hence forth termed “BDNF”) (Foltran and Diaz, 2016; Kowianski et al., 2018; Figure 2). The pro domain has been identified as an independent ligand itself and encodes the single nucleotide polymorphism of methionine to valine substitution at position 66 in the BDNF gene (Egan et al., 2003; Dieni et al., 2012; Notaras and van den Buuse, 2018). Intracellular cleavage of pro-BDNF in the TGN occurs via furin, while its cleavage to form BDNF in secretory vesicles requires convertases. The final molecular weight of BDNF is 14 kDa, consisting of 119 amino acids (Lu et al., 2005). The pro-BDNF is also secreted extracellularly, and then cleaved by proteases such as plasmin and metalloproteinases (MMP2 and MMP9) to form BDNF (Hwang et al., 2005; Mizoguchi et al., 2011). The extracellularly secreted pro-domain, pro-BDNF and BDNF are all biologically active and perform their various physiological functions (Figure 2).

The packaging and secretion of pro domain, pro-BDNF and BDNF from within the TGN into dense core secretory vesicles occurs via the constitutive secretory pathway and a preferential tightly controlled regulated pathway (Goodman et al., 1996; Lu, 2003; Brigadski et al., 2005). BDNF is secreted both pre- and post-synaptically, and undergoes anterograde and retrograde transport via autocrine and paracrine mechanisms (Cunha et al., 2010). These mechanisms modulate synaptic transmission and synaptogenesis (Cunha et al., 2010) via Ca^2+^-dependent mechanisms. BDNF is secreted pre-synaptically via increased intracellular influx of Ca^2+^ (Balkowiec and Katz, 2002). Post-synaptically, the secretion of BDNF is by regulated activity-dependent increases in Ca^2+^, entering via ionotrophic glutamate receptors and voltage-gated Ca^2+^ channels (Hartmann et al., 2001), or Ca^2+^ release from intracellular stores (Griesbeck et al., 1999) and release occurs via endosome like vesicles where exogenous BDNF is recycled (Sasi et al., 2017). Altogether, the above described synthesis, processing and secretion of BDNF gives rise to three functionally active proteins: the pro domain of BDNF, pro-BDNF and BDNF (mature BDNF) (Figure 2; Hempstead, 2015). Once released, they interact with their respective receptors to exert their distinct physiological functions.

## BDNF Isoforms and Their Receptors

The three products of the BDNF gene bind to specific receptors and regulate distinct biological functions. The pro-domain of BDNF binds to sortilin, a member of vacuolar protein sorting 10 protein (vps10p) of the sorting receptor family (Teng et al., 2005; Anastasia et al., 2013) to trigger specific functions in developing and adult neurons. The pro-domain acts by inducing growth cone retraction (Anastasia et al., 2013), facilitating long term synaptic depression (LTD) in developing neurons (Mizui et al., 2016), and modulating synaptic spine density and neuronal network plasticity via a cytochrome c caspase-3 mechanism in adult neurons (Guo et al., 2016). The pro-BDNF, comprising of the pro-domain and mature domain, act via preferential interactive binding to p75, a member of the tumor necrosis factor receptor family, and sortilin receptors, respectively (Teng et al., 2005; Kowianski et al., 2018) and with lower affinity binding to TrkB. The binding of pro-BDNF/p75/sortilin initiates the activation of c-Jun amino terminal kinase (JNK), Ras homolog gene family member A (RhoA), and nuclear factor kappa B (NF-κB) cascade (Reichardt, 2006; Anastasia et al., 2013; Kowianski et al., 2018). These signaling cascades (JNk, Ras, and NF-κB) in turn trigger a number of diverse cellular and morphological outcomes, such as neuronal apoptosis (Teng et al., 2005), neuronal growth cone development, and neuronal survival (Reichardt, 2006).

The third product, BDNF, binds with high affinity to TrkB of the Trk family of tyrosine kinases and with lower affinity to the p75 receptor (Chao and Hempstead, 1995; Reichardt, 2006). Activation of these two receptors is responsible for BDNF’s known functions. In brief, BDNF/TrkB activation aids in neurogenesis, gliogenesis, neurite outgrowth, and enhanced neuronal survival (Huang and Reichardt, 2001; Vilar and Mira, 2016). In developing neuronal circuits, BDNF acts to regulate dendritic arborization and spine formation (Deinhardt and Chao, 2014; Gonzalez et al., 2016), and enhances long term synaptic potentiation (LTP) (Park and Poo, 2013; Leal et al., 2015). In mature neurons, BDNF is also required to sustain viability (Alcantara et al., 1997). BDNF mediates opposing actions on binding to the p75 receptor; while BDNF/TrkB enhances neuronal excitability and synaptic strength, BDNF/p75 acts to decrease excitability and synaptic strength and induce neuronal plasticity (Sasi et al., 2017), initiating JNK (Reichardt, 2006; Anastasia et al., 2013; Kowianski et al., 2018), triggering neuronal apoptosis (Teng et al., 2005), and NF-κB cascade regulating of neuronal growth cone development and navigation and neuronal survival. The TrkB and p75 receptor have somadendritic distribution (Bronfman and Fainzilber, 2004), where TrkB is localized to the pre- and post-synaptic membranes and intracellularly (Gomes et al., 2006; Song et al., 2017).

Brain derived neurotrophic factor undergoes slow exocytosis (Brigadski et al., 2005) following depolarization and stimulation of glutamate receptors (Righi et al., 2000; Kohara et al., 2001). Thus, activity-dependent BDNF secretion can be induced by numerous stimuli including high potassium, glutamate and the neurotrophin itself, dependent on intracellular Ca^2+^ increase (Blochl and Thoenen, 1995; Goodman et al., 1996; Canossa et al., 1997).

### The TrkB Receptor

The TrkB receptor is encoded by a single TrkB gene, the NTRK2 gene encoding 24 exons located on chromosome 9q22 (Schneider and Schweiger, 1991; Nakagawara et al., 1995). TrkB consists of three domains: – an extracellular ligand binding domain, a transmembrane domain and an intracellular tyrosine kinase domain. One full- length TrkB (TrkB) contains an extracellular transmembrane domain, consisting of a cysteine rich cluster followed by 3 leucine repeats, a cysteine cluster followed by 2 immunoglobulin (Ig1 and Ig2) domains; and an intracellular cytoplasmic tyrosine kinase domain acting as a phosphorylation dependent docking site (Schneider and Schweiger, 1991; Tejeda and Diaz-Guerra, 2017). The Ig domain in exon 12 directs binding specificity to its ligand, BDNF. Exon 15 encodes the transmembrane domain, and exon 20–24 the intracellular tyrosine kinase domain (Middlemas et al., 1991). The first five exons serve as the transcription initiation sites and display alternate splicing patterns (Stoilov et al., 2002). Exon five also serves as a ribosomal entry site, directing the start of translation and producing four isoforms of TrkB receptors in humans (Luberg et al., 2010; Sasi et al., 2017). Other splice variants are two truncated TrkB (TrkB-T1) isoforms, TrkB-T2, TrkB- Shc lacking tyrosine kinase domain, and a TrkB-TK with a non-viable catalytic domain. Truncated TrkB receptors (TrkB-T1 and TrkB-T2) are the product of alternate splicing at exon 18. These TrkB isoforms are activated on binding to BDNF to initiate downstream signaling.

### BDNF/TrkB

Brain derived neurotrophic factor binds to the TrkB receptor, both TrkB-T and TrkB (full length) with similar affinity (Sasi et al., 2017). Exactly how TrkB receptor isoforms coordinate and produce a precise cellular and biological function is not yet clearly understood. BDNF binding to the TrkB-T isoform has been identified as a dominant negative pathway (Fenner, 2012; Notaras and van den Buuse, 2018). More recently, the TrkB-T receptor has also been reported to have functions apart from dominant negative regulation, including the following: metabolite release (Baxter et al., 1997; Fenner, 2012); BDNF sequestration and translocation (Fryer et al., 1996); filopodia and neurite outgrowth (Fryer et al., 1997; Yacoubian and Lo, 2000; Fenner, 2012); and astrocytic cytoskeletal remodeling via Rho GTPase (Ohira et al., 2006; Fenner, 2012). Additionally, alterations in expression of TrkB-T has also been shown to alter neuronal viability, resulting in neurodegeneration (Vidaurre et al., 2012), indicating a biological function of TrkB-T. However, the mechanism driving the function of TrkB-T is not well understood. Given that this review focusses on the interaction of BDNF with the TrkB receptor, the following sections will only address BDNF-TrkB interactions together, independent of BDNF’s non-TrkB functions.

**FIGURE 3:**
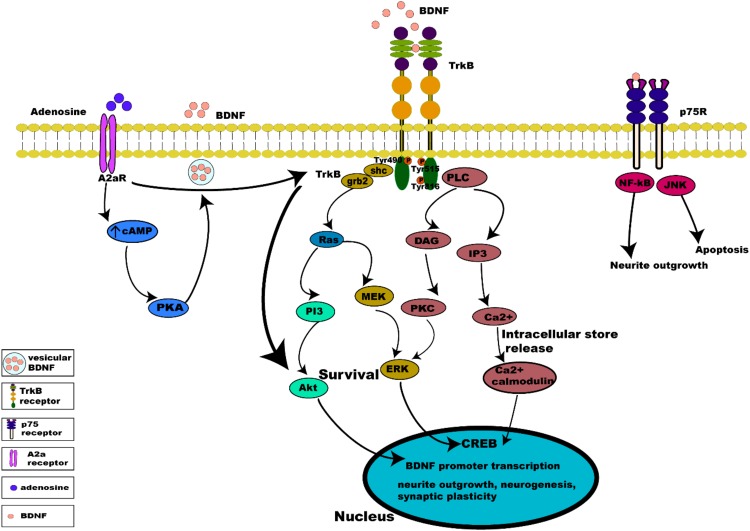
Intrinsic intracellular signaling cascades of BDNF-TrkB and TrkB-A_2__*a*_ receptors. BDNF-TrkB activation predominantly initiates MAPK, PI3K, and PLC γ signaling pathways. Activation of the TrkB receptor at its Tyr490 and Tyr515 residue recruits Shc adaptor protein leading to binding of growth factor receptor bound protein 2 (grb2) which binds with GTPase Ras to form a complex and initiate extracellular signal regulated kinase (ERK) activation which in turn activates the mitogen activated protein kinase MAPK/ERK pathway, which results in the activation of CREB transcription factor. Activation of Tyr515 residue also activates the PI3K signaling pathway, incorporating combined actions of Ras and activating the PI3/Akt and MEK/MAPK pathways. Both MAPK and PI3K signaling exert neurotrophic functions of survival, growth and differentiation, via activation transcription factors (CREB and C-myc). Phosphorylation of the TrkB receptor at its Tyr816 residue activates the phospholipase C γ (PLC γ) pathway, generating inositol-1, 4, 5-triphosphate (IP3) and diacylglycerol (DAG). The PLCγ/IP3 pathway results in calcium release from intracellular stores, in turn activating Ca^2+^/CaMKII. DAG activates PKC, leading to synaptic plasticity. pro-BDNF/p75 initiates JNK signaling (Reichardt, 2006; Anastasia et al., 2013; Kowianski et al., 2018) triggering neuronal apoptosis (Teng et al., 2005), and the NF-κB signaling cascade regulation of neuronal growth cone development and navigation, and neuronal survival. Like BDNF, neuronal activity promotes release of adenosine which binds to A_2__*a*_Rs to activate adenylyl cyclase leading to production of cAMP further activating PKA downstream which controls Ca^2+^ dependent BDNF release. Activation of A_2__*a*_Rs also transactivates TrkB, initiating the TrkB-Akt pathway promoting neuronal survival.

Binding of BDNF to TrkB initiates two different categories of cascades: a fast acting BDNF/TrkB cascade that excites neurons or a slow acting occurring over minutes to hours. The action of BDNF markedly differs between these two categories (Kafitz et al., 1999; Ji et al., 2010). Additionally, evidence suggests that the well-studied effect of BDNF/TrkB on cell survival and plasticity are mediated by TrkB-FL (Klein et al., 1993; Carim-Todd et al., 2009). Once BDNF binds to TrkB, ligand-mediated dimerization of the complex occurs at the cell surface, followed by autophosphorylation of specific tyrosine residues in the cytoplasmic domain, leading to activation of three interconnected intrinsic intracellular cascades (Chao, 2003; Cunha et al., 2010; Figure 3).

## The MAPK Pathway

Activation of the TrkB receptor at its Tyr490 and Tyr515 residue causes the docking of Shc adaptor protein (Src- homology 2-domain) at these tyrosine sites and recruits growth factor receptor bound protein 2 (grb2) which binds with GTPase Ras to form a complex, and initiates extracellular signal regulated kinase (ERK) activation (Wheaton et al., 2007). ERK activation in turn activates the mitogen activated protein kinase MAPK/ERK pathway. MAPK/ERK kinases are able to phosphorylate and activate the transcription factor cAMP response element binding protein (CREB) (Huang and Reichardt, 2003; Begni et al., 2017). The phosphorylated CREB is then translocated into the nucleus, where it induces BDNF transcription by binding to BDNF promoters (Shaywitz and Greenberg, 1999). Binding to BDNF promoters drives BDNF expression to regulate neuronal survival, differentiation and synaptic plasticity (Patapoutian and Reichardt, 2001). In addition, this activation of BDNF expression induces activation of AMPA receptors on stimulation by BDNF (Song et al., 2013).

## The PI3K Pathway

Activation of the PI3K pathway incorporates combined actions of Ras at the Tyr515 residue, which activates multiple cascades, namely the PI3/Akt and MEK/MAPK pathways. Activation of the PI3K/Akt cascade regulates proteins such as BAD (Bcl-2 antagonist of cell death) and GSK-3β (glycogen synthase kinase 3β), essential for neuronal survival, growth and differentiation, and is activated by Ca^2+^ influx via L-type voltage gated calcium channels (L-VGCC) (Brunet et al., 2001). Activation of mammalian target of rapamycin (mTOR) by BDNF also enhances local BDNF translation to dendrites at active synapses via the PI3K pathway (Schratt et al., 2004).

## The PLC γ Pathway

The phosphorylation of the TrkB receptor at its Tyr816 residue activates the phospholipase C γ (PLC γ) pathway, generating inositol-1,4,5-triphosphate (IP_3_), and diacylglycerol (DAG) which is important for survival, neurite outgrowth and synaptic plasticity. BDNF via the PLCγ/IP_3_ pathway results in calcium release from intracellular stores activating CaMKII (Ca^2+^/calmodulin dependent protein kinase) which in turn activates CREB phosphorylation (Minichiello et al., 2002; Tejeda and Diaz-Guerra, 2017). The generation of DAG on the other hand, activates PKC (Bellingham, 2013a) which is translocated to the membrane for further activation and phosphorylation of ERK leading to synaptic plasticity (Minichiello et al., 2002; Chao, 2003).

The BDNF-TrkB complex not only activates on its transmembrane surface (described above) but it also internalizes via endosomes (both early and late endosomes) to activate downstream pathways. This BDNF-TrkB signaling via endosome also determines the cellular fate of BDNF-TrkB complexes, which can be transported retrogradely, recycled back to the membrane, or prepared for degradation by lysosomes (Yamashita and Kuruvilla, 2016).

## BDNF: Role in Synaptic Transmission

Synaptic transmission is a highly complex trans-neuronal process, occurring at the synapse between a pre-synaptic (axonal) terminal and a post-synaptic (typically dendritic) membrane. BDNF elicits rapid effects on synaptic transmission and membrane excitability, via activation of pathways in both the pre- and post-synaptic compartments. In the pre-synaptic compartment, BDNF causes release of glutamate and GABA, via the TrkB-ERK mediated pathway (Jovanovic et al., 2000). Enhanced glutamate release at glutamatergic synapses is mediated by an increase in docked vesicles at presynaptic active zones (Tyler and Pozzo-Miller, 2001). For example, BDNF application to hippocampal and cortical neuron cultures (Levine et al., 1995; Lessmann, 1998) and slice preparations (Kang and Schuman, 1995; Kang et al., 1997) potentiates excitatory neurotransmission, increasing glutamate release. Consequently, BDNF application onto brain slices induces hyper-excitability (Scharfman, 1997), which is consistent with observations in transgenic mice over-expressing BDNF (Croll et al., 1999). In the post-synaptic compartment, BDNF can also enhance synaptic responses by increasing the open probability of NMDA glutamate receptors (Rose et al., 2004). Hence, in the context of ALS, the increased neuronal activity observed in hSOD1^*G*93*A*^ mice is capable of increasing BDNF secretion, which in turn can increase release of glutamate to trigger excitotoxicity, leading to MN degeneration. Indeed, BDNF has been shown to enhance MN death by glutamate excitotoxicity, via activation of TrkB (Hu and Kalb, 2003; Mojsilovic-Petrovic et al., 2006). Together, these observations highlight a possible role for BDNF in the death of MNs in ALS.

## Mechanisms of BDNF-Modified Neurotransmission

Brain derived neurotrophic factor also modifies neurotransmission by altering the expression of pre-synaptic proteins that regulate neurotransmitter release (Andreae and Burrone, 2018). For example, in BDNF deficient mice, decreased synaptic transmission correlates with a drop in the number of docked synaptic vesicles (Carter et al., 2002). This is also correlated with decreases in synapsin, synaptophysin and synaptobrevin – presynaptic proteins required for vesicle docking and exocytosis at release sites (i.e., active zones) (Martinez et al., 1998; Pozzo-Miller et al., 1999; Jovanovic et al., 2000). These physiological and molecular changes are also present in TrkB knockout mice (Martinez et al., 1998). Thus, BDNF can stimulate synaptic transmission via three mechanisms: (1) increasing the number of synaptic vesicles at the active zone, (2) increasing the postsynaptic receptor response, and (3) increasing the overall number of synapses per neuron (Bradley and Sporns, 1999).

## TRKB Receptor Can Be Activated Independent of BDNF

Tropomysin related kinase B receptor is capable of autophosphorylation and activation of downward cascades independent of its ligand, BDNF. Activation of TrkB receptors in the absence of BDNF occurs via a mechanism known as trans-activation, which involves specific G protein coupled receptors (GPCR), such as the A_2__*a*_ adenosine receptor (A_2__*a*_R) present both pre- and post-synaptically (Chao, 2003; Sebastiao et al., 2018). Adenosine is a key neuromodulator produced both extracellularly and intracellularly in neurons and glial cells (Moreau and Huber, 1999). Extracellularly, it is produced by ectonucleotidase degradation of ATP released by neurons and astrocytes, and intracellularly by production during breakdown of ATP during high energy demand, followed by transport into the extracellular space (Jacobson and Gao, 2006). Adenosine directly regulates synaptic transmission and plasticity, as well as modulating neurotransmission and neurotrophins (Sebastiao and Ribeiro, 2009). Pre-synaptically the activation of A_2__*a*_Rs increases the release of glutamate (Ciruela et al., 2010; Cunha, 2016) and the activation of NMDA receptors (Azdad et al., 2009; Higley and Sabatini, 2010; Sarantis et al., 2015), thus facilitating LTP. Transactivation of TrkB by A_2__*a*_Rs is mediated by the Src family of protein, such as Fyn (Lee and Chao, 2001), in a slow acting cascade occurring over minutes to hours. TrkB/A_2__*a*_R interaction allows transactivation of a downstream protective TrkB-Akt pathway (Mojsilovic-Petrovic et al., 2006). Post-synaptic activation of A_2__*a*_Rs also triggers calcium dependent processes, through L-type voltage gated calcium channels and NMDA receptors, activating adenylyl cyclase and leading to increased cAMP and PKA phosphorylation, which in turn influences Ca^2+^ dependent transcription of BDNF mRNA (Zheng et al., 2011) and BDNF secretion (Tebano et al., 2010) (summarized in Figure 3).

A study by Diogenes et al. (2004), demonstrated that BDNF alone without prior depolarization was devoid of effects on neurotransmission, while enhancement of synaptic transmission by BDNF in the hippocampus was facilitated by pre-synaptic activity-dependent adenosine release via A_2__*A*_Rs. This excitatory action of BDNF can be blocked by a TrkB inhibitor, an A_2__*A*_R antagonist or by a PKA inhibitor, thus indicating that activation of A_2__*A*_Rs facilitates BDNF modulation of synaptic transmission (Diogenes et al., 2004). Additionally, the role of A_2__*a*_Rs in regulating BDNF function was further supported in a study using A_2__*a*_R KO mice, which showed no increase in field EPSCs after BDNF application, whereas in normal hippocampus slices BDNF induced enhanced field EPSCs and EPSCs, and this effect was blocked by A_2__*a*_R blockers, clearly indicating that activation of A_2__*a*_Rs is required for normal BDNF levels and BDNF’s potentiation of synaptic transmission (Tebano et al., 2008). Taking into account the enhanced glutamate present in MNs in ALS, the A_2__*a*_Rs is considered as a potential neuroprotective therapeutic agent to ameliorate glutamate induced excitotoxicity in ALS, reinforcing the significance of TrkB transactivation.

In addition to TrkB transactivation by A_2__*a*_Rs, GPCR mediated TrkB transactivation also occurs via other mechanisms. For example, in embryonic cortical neurons TrkB is transactivated by activation of epidermal growth factor (EGF) leading to migration of early cortical neurons to form a differentiated cortical layer (Puehringer et al., 2013). Similarly in striatal neurons, activation of dopamine 1 (D1) receptors leads to transactivation of TrkB functioning in axonal growth and growth cone during neuronal development (Iwakura et al., 2008). Furthermore, in hippocampal mossy fiber neurons TrkB is transactivated by zinc, which is secreted along with glutamate in response to neuronal activity leading to potentiation of mossy fiber synapses, thus regulating synaptic plasticity (Huang et al., 2008). Hence, considering the role of BDNF/TrkB during development and pathological situations such as in neurodegenerative diseases, TrkB transactivation offers alternative methods to modulate BDNF/TrkB, opening new therapeutic avenues for the treatment of neurodegenerative disorders.

## BDNF/TrkB Interacts With Ca^2+^ and Glutamate

The interplay between BDNF and glutamate has been well established in many previous studies. Glutamate is a major excitatory neurotransmitter in the central nervous system (CNS) known for its activity-dependent interplay with neurotrophic factors during development and in mature neurons. Post-synaptically, the effect of glutamate is mediated by activation of two major ionotrophic glutamate receptors; AMPA receptors and NMDA receptors (Rao and Finkbeiner, 2007; Mattson, 2008). Pre-synaptic depolarization results in glutamate release, activation of AMPA and NMDA receptors post-synaptically, and secretion of BDNF in the extracellular space (Nagappan and Lu, 2005). BDNF-induced pre-synaptic glutamate release is mediated via TrkB-ERK signaling (Jovanovic et al., 2000), and post-synaptic modulation of glutamate receptors occurs by phosphorylation of the NMDA receptor subunit NR2B (Cunha et al., 2010). Furthermore, BDNF also enhances AMPA receptor surface expression, thus increasing post-synaptic responses to glutamate (Narisawa-Saito et al., 2002; Cunha et al., 2010) – an effect mediated via ERK (Li and Keifer, 2009). BDNF treatment also leads to phosphorylation of NMDAR subunit NR1 (Slack and Thompson, 2002), altering NMDAR localization at synapses (Gomes et al., 2006).

In addition to these pre- and postsynaptic effects on glutamatergic transmission, TrkB activation also modulates ion channels that can alter neuronal excitability, including Na^+^, Ca^2+^, and K^+^ channels through intracellular cascades (Blum et al., 2002; Tucker and Fadool, 2002). For example, BDNF/TrkB activation alters neuronal excitability by gating of Na^+^ current via Nav1.9 (Blum et al., 2002). Metabotropic receptors such as A_2__*a*_Rs also activate TrkB to induce release of intracellular Ca^2+^ from ER stores. This in turn activates a PLC cascade to generate inositol triphosphate (IP3) which releases Ca^2+^ from IP3-sensitive stores, activating PKC.

**FIGURE 4:**
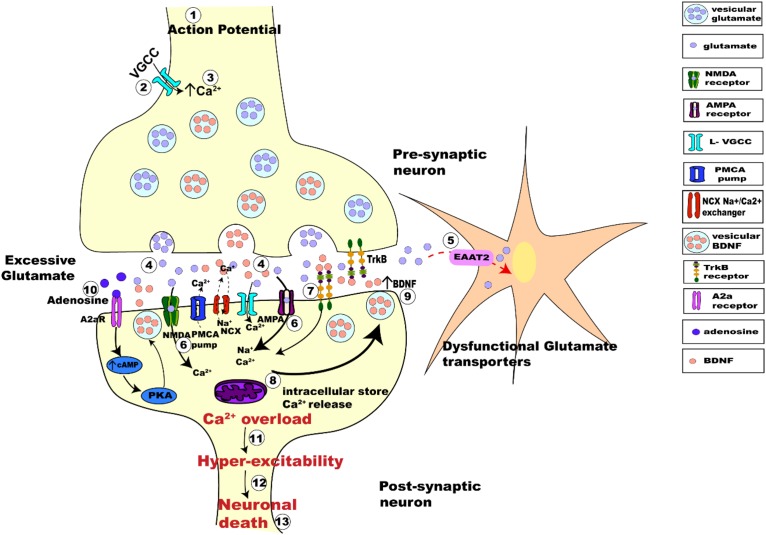
Schematic figure representing activity-dependent interplay between glutamate-induced excitotoxicity and BDNF-TrkB signaling. The arrival of action potential **(step 1)**, triggers the influx of Ca^2+^ ions into pre-synaptic terminal **(step 2)**, the rise in intracellular calcium **(step 3)** in turn triggers the fusion of synaptic vesicles with pre-synaptic membrane to release glutamate (light purple dots) from the terminal **(step 4)**. Membrane depolarization also results in BDNF secretion (orange dots) **(step 4)** and release pre-synaptically into the synaptic cleft. Dysfunctional glutamate transporters (EAAT2) in ALS results in retention of excessive glutamate in the synaptic cleft **(step 5)**. Post-synaptically, Ca^2+^ enters through voltage gated calcium channels and via calcium-permeable glutamate receptors (NMDA, green; AMPA magenta; **step 6**). BDNF binding to TrkB alters the neuronal excitability of ion channels and also enhances post-synaptic glutamate receptor activation causing influx Ca^2+^ of ions post-synaptically **(step 7)**. Excessive glutamate in the synaptic-cleft over-activates its receptors increasing the intracellular Ca^2+^ furthermore **(step 8)**. Impaired Na^+^/Ca^2+^ exchanger and ATP pumps in SOD1^*G*93*A*^ mice results in enhanced Ca^2+^ intracellularly (DeJesus-Hernandez et al., 2011; Sirabella et al., 2018). Ca^2+^ released from the intracellular store adds to the Ca^2+^ concentration **(step 8)**. Enhanced Ca^2+^ in the post-synaptic neuron also triggers increased Ca^2+^ dependent secretion and release of BDNF extracellularly causing enhanced BDNF extracellularly **(step 9)**. *Trans*-activation of TrkB by A_2__*a*_Rs also triggers calcium dependent signaling, activating adenylyl cyclase and leading to increased cAMP and PKA phosphorylation, which in turn influences BDNF secretion **(step 10)**. This increased BDNF in the synaptic cleft binds to its receptors TrkB and repeats the process of Ca^2+^ influx and modulation of glutamate receptors, eventually leading to Ca^2+^ overload **(step 11)** making the neurons hyper-excitable **(step 12)** which eventually leads to neuronal death **(step 13)**.

Considering the interplay between the actions of BDNF and glutamate, there are several possible avenues leading to interactions between neuronal activity and BDNF. Synaptic hyper-excitability and increased intrinsic excitability of susceptible neurons in ALS are clearly observed in human patients (Bostock et al., 1998; Mogyoros et al., 1998; Kanai et al., 2006; Sirabella et al., 2018) and in animal models of ALS (van Zundert et al., 2008; Fogarty et al., 2015; Sirabella et al., 2018). The increased firing and synaptic activation of glutamate receptors would likely result in increased intracellular Ca^2+^, enhancing BDNF release, which could trigger further release of glutamate. This proposed mechanism would perturb the neuron’s ability to regulate its activity, leading to glutamate excitotoxicity and neuronal death (Figure 4).

### BDNF/TrkB Crosstalk With Inhibitory Neurotransmission

Considering that the physiological functioning of neurons requires a balance between excitatory and inhibitory neuro-transmission, hyper-excitability of MNs in ALS can also result from reduced inhibition. γ-aminobutyric acid (GABA) and glycine are the primary neurotransmitters regulating chloride (Cl^–^) mediated inhibition in CNS by binding to their post-synaptic receptors. The strength of synaptic inhibition critically depends on intracellular Cl^–^ concentration, hence on Cl^–^ homeostasis (Rivera et al., 1999). A key regulator of Cl^–^ concentration, the potassium chloride cotransporter (KCC2) (Rivera et al., 1999) has been shown to be depleted in MNs (Boulenguez et al., 2010; Fuchs et al., 2010), contributing to the hyper-excitability of MNs. Furthermore, BDNF/TrkB has been shown to be associated with KCC2 regulation (Lee-Hotta et al., 2019) and BDNF/TrkB dependent KCC2 depletion has also been described in MNs (Fuchs et al., 2010; Lee-Hotta et al., 2019). Thus, BDNF/TrkB activation down regulates the expression of KCC2 thereby reducing the Cl^–^ extrusion capacity in MNs (Rivera et al., 2002, 2004), suppressing Cl^–^ dependent inhibition which as a result makes the neuron hyper-excitable. Also, the role of microglia induced synaptic inhibition cannot be ignored because KCC2 modulation is required to achieve synaptic balance (Fiumelli and Woodin, 2007; Ferrini and De Koninck, 2013). Indeed, BDNF is released not only from neurons but also from microglia, making BDNF/TrkB a major signaling point of interaction between microglia and neuron (Trang et al., 2011), eventually affecting Cl^–^ homeostasis (Rivera et al., 2002, 2004; Coull et al., 2005; Zhang et al., 2008). Besides, microglial activation and dysfunction observed in MNs of ALS mice contributes to the progression of disease (Brites and Vaz, 2014) making microglial BDNF a potential actor.

## Detrimental Effects of BDNF/TrkB in ALS

The ability of BDNF/TrkB to promote neuronal survival and resistance to toxic insults is well characterized (Kowianski et al., 2018). Several studies documented the neuroprotective effects of BDNF on glutamate induced excitotoxicity *in vivo* (Bemelmans et al., 2006; Henriques et al., 2010) and functional recovery of motor neurons *in vitro* following exogenous BDNF application (Shruthi et al., 2017). Contrary to the view stated in this review, potentiating BDNF has been one of the strategies to delay the disease progression of ALS. The modulation of TrkB via small molecule drug formulations to enhance BDNF signaling also enhanced neuronal survival in degenerating neurons *in vitro* (Guerzoni et al., 2017) and improved motor function and motor neuron loss in ALS model mice (Korkmaz et al., 2014). BDNF potentiation has also been demonstrated to enhance MN survival *in vitro* (Tsai et al., 2013) and in other neurodegenerative diseases (Aytan et al., 2018). Additionally, transactivating TrkB by A_2__*a*_ receptors has also been reported to enhance survival of MNs in culture (Komaki et al., 2012). However, despite these neuroprotective effects observed there is also evidence to show that therapeutic interventions aimed at enhancing BDNF/TrkB are unable to promote survival or prevent death of neurons *in vivo* (The BDNF Study Group Phase III, 1999; Silani et al., 2001; Pansarasa et al., 2018). This suggests that the detrimental actions of BDNF also need to be considered. Under certain circumstances, many studies report that BDNF/TrkB can exert negative effects on MN survival, making MNs more vulnerable to insults (Hu and Kalb, 2003; Mojsilovic-Petrovic et al., 2006). Moreover, BDNF is potent at enhancing excitotoxic insult, by enhancing glutamatergic activity in neurons (Kafitz et al., 1999). Several studies report BDNF and TrkB to be key players in rendering MNs vulnerable to excitotoxic insult (Fryer et al., 2000; Hu and Kalb, 2003; Mojsilovic-Petrovic et al., 2005). Additionally, muscle from ALS patients expresses elevated levels of BDNF, suggesting the possible negative action of BDNF (Kust et al., 2002). Furthermore, BDNF can accelerate glutamate-induced death in rat neuroblastoma cells, and this effect was promoted by TrkB activation (Maki et al., 2015). BDNF also elicited glutamate excitotoxicity in cultured cortical neurons (Koh et al., 1995; Kim et al., 2003), and TrkB inhibition ameliorated these detrimental effects of BDNF (Kim et al., 2003). Additional evidence for the role of BDNF/TrkB in promoting neuronal death comes from a study, where exogenous nitic oxide (NO)/sodium nitroprusside-induced cell death in cortical neurons was enhanced by BDNF, and this effect was inhibited by TrkB antagonism (Ishikawa et al., 2000). Fryer et al. (1999, 2000) also demonstrated that BDNF enhanced glutamate excitotoxicity in cultured embryonic spinal cord MNs, and this response involved activation of TrkB. Furthermore, directly blocking TrkB activation protected embryonic cultured MNs from toxic insults thought to be involved in the pathogenesis of ALS, such as excitotoxicity and the presence of SOD1 mutations (Hu and Kalb, 2003; Mojsilovic-Petrovic et al., 2006; Jeong et al., 2011). Additionally, TrkB-T receptors have been shown to be enhanced in MNs in ALS and deletion of TrkB-T receptors delayed the progression of disease in SOD1^*G*93*A*^ mice (Yanpallewar et al., 2012) which further adds to the role of TrkB in ALS. Similarly, impaired BDNF/TrkB signaling and altered TrkB-T isoform was observed in the neuromuscular junction of pre-symptomatic SOD1^*G*93*A*^ mice (Just-Borras et al., 2019). Furthermore, removal of the TrkB-T receptor at the pre-symptomatic stage in SOD1^*G*93*A*^ mice improved the disease symptoms rescuing hippocampal interneurons and regulating long term potentiation (Quarta et al., 2018) found to be enhanced in ALS (Spalloni et al., 2006).

In addition to the above BDNF-TrkB signaling effects, blocking A_2__*a*_Rs, which co-localize with and directly transactivate TrkB, protected cultured MNs from these detrimental effects (Mojsilovic-Petrovic et al., 2005, 2006; Ng et al., 2015; Cunha, 2016). Similarly the inhibition of TrkB or A_2__*a*_Rs also prevents toxicity following expression of the G85R or G37R SOD1 mutations, which are highly toxic to cultured MNs (Mojsilovic-Petrovic et al., 2006; Jeong et al., 2011). These pro-toxic effects of BDNF/TrkB are not merely an artifact of culturing embryonic MNs. It has also been shown that *in vivo* conditional deletion of TrkB in MNs of adult transgenic mice carrying a G85R SOD1 mutation attenuates SOD1 toxicity, resulting in extension of life span and motor function, slowing MN loss and causing persistence of neuromuscular junctions (Zhai et al., 2011). Furthermore, in a recent study utilizing SOD1^*G*93*A*^ rats, phrenic motor neurons displayed an increased expression of BDNF and phosphorylated ERK at end stage disease, consistent with possibly increased BDNF function and basal protein levels (Nichols et al., 2017).

Hyper-activity induced activation of BDNF and TrkB have also been observed in other disease states, such as epilepsy and traumatic brain injury (Dai et al., 2010; Iughetti et al., 2018). Upregulated expression of BDNF and TrkB has been well documented, resulting in alteration of excitability and neuronal network activity contributing to epileptogenesis (Scharfman, 1997; Iughetti et al., 2018). Enhancing BDNF expression or its systemic administration enhanced seizure activity in mice (Croll et al., 1999; Scharfman et al., 2002; Iughetti et al., 2018), while inhibiting TrkB reduced seizure development in these animals (Heinrich et al., 2011; Liu et al., 2014; Iughetti et al., 2018). Additionally, genetic or pharmacological inhibition of A_2__*a*_Rs in epilepsy has been shown to reduce seizures and neuronal damage (El Yacoubi et al., 2008, 2009). Despite these numerous reports, the concept of hyper-activity induced glutamate excitotoxicity resulting in overexpression of BDNF and TrkB activation in neuronal death still needs further investigation.

## Conclusion

Amyotrophic lateral sclerosis is an incurable multi-factorial disease state where synaptic and intrinsic hyper-activity of MNs is a significant early factor (Mogyoros et al., 1998; Kanai et al., 2006; van Zundert et al., 2008; Fogarty et al., 2015). Therapeutic avenues until now have aimed at a reduction of this excitable state. Neuronal hyper-activity is plausibly a result of processes that take place simultaneously, one of them being the secretion of BDNF and activation of its receptor TrkB. Several lines of evidence show that increased BDNF-TrkB is evident in a number of neurodegenerative diseases, including ALS (Kust et al., 2002; Hu and Kalb, 2003; Nichols et al., 2017). This suggests that neuronal damage may be a result of excessive rather than a shortage of, neurotrophic support. A broader understanding of the factors that regulate altered neuronal activity and BDNF could help to identify new therapeutic targets in neurodegenerative diseases. Certainly, therapies that enhance endogenous BDNF have failed to produce any success in prevention or slowing of MN death in ALS. It is important to further investigate both pro- and anti-trophic functions of BDNF/TrkB in the hope of discovering novel therapeutic avenues to alleviate this devastating disease and other neurodegenerative conditions.

## Author Contributions

All authors contributed to the writing and editing of the manuscript.

## Conflict of Interest Statement

The authors declare that the research was conducted in the absence of any commercial or financial relationships that could be construed as a potential conflict of interest.
